# Apatinib affect VEGF-mediated cell proliferation, migration, invasion via blocking VEGFR2/RAF/MEK/ERK and PI3K/AKT pathways in cholangiocarcinoma cell

**DOI:** 10.1186/s12876-018-0870-3

**Published:** 2018-11-06

**Authors:** Manping Huang, Bin Huang, Guowen Li, Sainan Zeng

**Affiliations:** 1 Department of Intervention, Hunan Cancer Hospital & The Affiliated Cancer Hospital of Xiangya School of Medicine, Central SouthUniversity, No.283 , Tongzipo Road, Changsha, 410013 People’s Republic of China; 2 Infection Controlling Center, The Third Xiangya Hospital of Central South University, Tongzipo Road, Yuelu District, Changsha, 410013 People’s Republic of China

**Keywords:** Cholangiocarcinoma, Apatinib, VEGF, KDR (VEGFR2), RAF/MEK/ERK pathway, PI3K/AKT pathway

## Abstract

**Background:**

Cholangiocarcinoma (CCA) is a form of cancer that easily aggress to contiguous structures. Vascular endothelial growth factor (VEGF) and VEGF receptor 2 (VEGFR2) are increased in majority species of cancers and suppress tumor progression by blocking VEGF/VEGFR2. Apatinib is a highly selective VEGFR2 antagonist which has inhibitive effect on antiapoptotic and cell growth in CCA. While, the effect of apatinib cell migration and invasion in CCA is still unknown.

**Methods:**

CCA cell lines QBC939 and TFK-1 were transfected with siKDR to establish the KDR function loss cell model, and recombined human VEGF (rhVEGF) protein was added into the culture medium to enhance the VEGF expression. RT-qPCR and western bloting were used to detect the mRNA and protein expression levels of VEGFR2 to investigate whether it was effectively repressed or activated with rhVEGF or apatinib treatment. Then, MTT, wound healing assay, and transwell matrix assay were applied to measure the effect of apatinib and rhVEGF on cell viability, migration and invasion, respectively.

**Results:**

The mRNA and protein expressions of VEGFR2 were significantly reduced with KDR RNAi in both QBC939 and TFK-1 cells, and rhVEGF treatment increased these expression levels (*p* < 0.05). Apatinib dramatically suppressed VEGF-mediated cell migration and invasion at the concentration of 100 nM treatment and significantly decreased the expression of metastasis-associated protein such as Slug, snail and MMP9. Moreover, all of these inhibiting effects of apatinib depended on the VEGFR2 existence. In addition, VEGFR2/RAF/MEK/ERK and PI3K/AKT signal pathways were enhanced by the introduction of rhVEGF, but were dramatically suppressed after the apatinib treatment.

**Conclusion:**

Apatinib inhibit VEGF-mediated cell migration and invasion in CCA cell lines via inhibiting the VEGFR2/RAF/MEK/ERK and PI3K/AKT pathways. It will be a potentially effective targeted drug for CCA.

## Background

Cholangiocarcinoma(CCA), also known as bile duct cancer, is a form of cancer that originates in the epithelial cells of bile ducts, along intrahepatic and extrahepatic biliary tree, that is defined as intrahepatic, peri-hilar and distal CCA [4, 22]. Due to the high aggressive ability, CCA could easily infiltrate into adjacent organs such like liver, hepatic artery and portal vein [29]. The infiltration patterns of CCA were distributed in lymph, vascular infiltration site, and lymph node metastases, which is a basic feature of CCA [6, 12].

Vascular endothelial growth factor (VEGF), originally known as vascular permeability factor (VPF), is a signal protein produced by epithelial cells [23]. It has been identified as a key player in neovascularization and cell proliferation in a variety of cancers, including the fatal biliary CCA [5, 21]. Clinical data shows VEGF was significantly increased in the biopsy samples of CCA [2, 18, 26]. Furthermore, there is evidences that blocking VEGF/VEGFR2 pathway can effectively inhibit the proliferation, migration, invasion, survival and adhesion ability of hepatocellular carcinoma, hyperplastic cholangiocyte and non-small cell lung cancer [14, 32].

Apatinib, a tyrosine kinase inhibitor that selectively inhibits the vascular endothelial growth factor receptor-2 (VEGFR2, also known as KDR), could significantly inhibit intracellular VEGF signaling [28]. Benefiting from the blocking effect of VEGF pathway, apatinib play a prominent role in inhibiting tumor cells anti-apoptosis, cells proliferation in vitro and repressing the growth of xenograft tumor in vivo [19, 20]. In additon, apatinib reveals inhibition effect on migration and invasion in KIF5B-RET driven tumors therapy [13]. However, up to now there are currently few studies on the impact of CCA migration and invasion. In this study, we investigated the role of apatinib in CCA migration and invasion via the QBC939 and TFK-1 cell line. Moreover, we also explored the potential mechanism that the inhibition effect of apatinib may via VEGFR2/RAF/MEK/ERK and PI3K/AKT pathways.

## Methods

### Cell culture and transfection

Human CCA cell lines QBC939 and TFK-1 were purchased from Suer Biological Inc. (Shanghai, China). QBC939 cells were cultured in Dulbecco’s Modified Eagle Medium (DMEM, Sigma-Aldrich, St. Louis, MO, USA) and TFK-1 cells were cultured in RPMI-1640 medium (Gibco-BRL, Gaithersburg, MD), both supplemented with 10% heat-inactivated fetal bovine serum (Gibco; Thermo Fisher Scientific, Inc., Waltham, MA, USA), and incubated at 37 °C with 5% CO_2_.

Cells were sub-cultured to 6-well plates until the confluence reached 80%. The final concentration 50 mM siKDR and siControl (labeled with a fluorescent, synthesize by Gene Pharma, Suzhou, China) were diluted in serum-free MEM, and gently mixed with Lipofectamine 2000 (6 μl/well, Sigma-Aldrich, St. Louis, MO, USA) following 5 min stand, respectively. Before added this mixture to cells, another 20 min stand at room temperature is needed. The transfected cells incubated at 37 °C with 5% CO_2_ for 8 h, and then change the medium into McCoy’s 5A medium containing 10% FBS without antibiotics. 24 h post transfection, the transfection efficiency was checked by fluorescence detection.

### RT-qPCR

VEGFR2 mRNA levels from two group cells were tested by RT-qPCR. The first group cells were transfected with siKDR and siControl for 24 h. Cells in the second group was treated with 0, 20, 50, 100, and 200 ng/ml recombinant human VEGF (rhVEGF, PeproTech, 100–20-2) for 2 h. Cells were subsequently homogenized and centrifuged (12,000 x g, 10 min, 4 °C) using TRIzol reagent (Sigma-Aldrich, St. Louis, MO, USA) for total RNA extraction. RNA purity and concentration were determined by Nano-Drop (Thermo Scientific).

1 mg total RNA was reverse transcribed into cDNA using GoScript™ RT system (Promega, Madison, WI, USA). qPCR was performed in triplicate as: 95 °C for 30 s, 40 cycles of 95 °C for 5 s, 58 °C for 10 s and 72 °C for 30 s, subsequently analyze melting curve. GAPDH was used as the reference gene. Primers (forward, reverse) were: VEGFR2 5’-GGACTCTCTCTGCCTACCTCAC-3′, 5’-GGCTCTTTCGCTTACTGTTCTG-3′, GAPDH 5’-AGAAGGCTGGGGCTCATTTG-3′, reverse 5’-AGGGGCCATCCACAGTCTTC-3′. The relative fold change of VEGFR2 was calculated by 2^-ΔΔCt^ method.

### MTT assay

After QBC939 cells and TFK-1 cells were cultured to 96-well plates (1 × 10^5^ cells/well) overnight, three conditions of drug treatment were set: (1) cells treated with 0, 10, 100, 1000 and 10,000 nM apatinib (MCE, HY13342) for 24 h; (2) cells treated with 0, 20, 50, 100 and 200 ng/ml rhVEGF for 2 h; (3) cells treated with 100 ng/ml rhVEGF for 2 h following treated with 10, 100, 1000 and 10,000 nM apatinib for 24 h And then cells were cultured for another 24 h, 10 mg/ml MTT was added and incubated for further 4 h. After that, cells were centrifuged at 1,000×g for 5 min at room temperature, removed supernatant, and added 100 μl DMSO to each well for 30 min to dissolve the formazan product. The optical density (OD) was measured at 492 nm by a microplate reader (FLx800; BioTek, Winooski, VT, USA). The relative cell viability was normalized with control group using optical density values.

### Wound healing assay

Cells were cultured to 6-well plates (2 × 10^5^ cells/well) until about 100% confluence. 100 nM apatinib or 100 ng/ml rhVEGF + 100 nM apatinib were added into medium and cultured 24 h. 200 μl pipette tip was used to create a wound gap on cell monolayer, and Olympus IX71 microscope (Olympus Corporation, Tokyo, Japan) at 100 times magnification was used for imaging immediately. Migration was then observed 24 h post wound scratched. Image-Pro Plus software (Media Cybernetics, Inc., Rockville, MD, USA) was used to calculated the relative migration distant% as: [(The relative distance recorded at 0 h - the relative distance recorded at 24 h)/the relative distance recorded at 0 h] × 100.

### Transwell matrix assay

Control and siKDR transfected cells (1 × 10^4^ cells/well) were incubated into the top chamber of matrigel coated polyethylene terephthalate membrane (50 μl/well, Corning, Corning, USA), and 100 nM apatinib or 100 ng/ml rhVEGF were added into the upper chamber,. After culturing for 24 h, cells in the upper chamber were removed gentlyand the invaded cells left at the bottom of chamber were fixed with 4% paraformaldehyde for 30 min and then stained with 0.1% crystal violet for 30 min. Following by counting under an optical microscope (Olympus Corporation, Tokyo, Japan) at a magnification of 200.

### Western blotting

After cells treated with/without 100 ng/ml rhVEGF, 100 nM apatinib or 100 ng/ml rhVEGF + 100 nM apatinib, cells were lysed using lysis buffer (Cell Signaling Technology, Danvers, USA) to extract total protein. Protein lysates were separated by 10% SDS-PAGE, followed by transfer to nitrocellulose membranes. The membrane was then blocked with 5% milk diluted in PBS at room temperature for 1 h, followed by incubated with 1:1000 VEGFR2 antibody (ab10972, Abcam, Cambridge, MA, USA),1:5000 p-VEGFR2 (ab38473, Abcam), 1:2000 p-MEK (2338, CST), 1:1000 MEK (4694, CST), 1:2000 p-ERK1/2 (4370, CST), 1:1000 ERK (4695, CST), 1:2000 slug (ab51772, Abcam), 1:3000 Snail (ab53519, Abcam), 1:2500 MMP9 (ab38898, Abcam), 1:1500 P-AKT (ab81283, Abcam), 1:1500 AKT (ab179463, Abcam) and 1:5000 GAPDH antibody (ab8245, Abcam) overnight at 4 °C separately. Once primary antibodies were washed, membrane was incubated with goat anti-rabbit horseradish peroxidase-labeled secondary antibody (Sangon Biotech, Shanghai, China). Protein bands were detected by incubating the membrane with Western Bright enhanced chemiluminescence working solution (Advansta, Menlo Park, CA, USA). The film (Kodak XBT-1, Carestream, Xiamen, China) was scanned with Bio-rad Gel Doc XR+ (BIO-RAD, Shanghai, China).

### Statistical analyses

Statistical analysis was conducted with the Social Sciences software version 17.0. Quantitative data were presented as mean ± SD. The two-tailed Student’s t test was applied to analyze statistical differences between two groups. For multiple comparisons, the one-way ANOVA was used to analyze the difference. *p* < 0.05 was considered to be statistically significant. Each test data was repeated at least three times.

## Results

### RNA interference reduced VEGFR2 mRNA and protein levels in QBC939 and TFK-1 cells

**Fig. 1:**
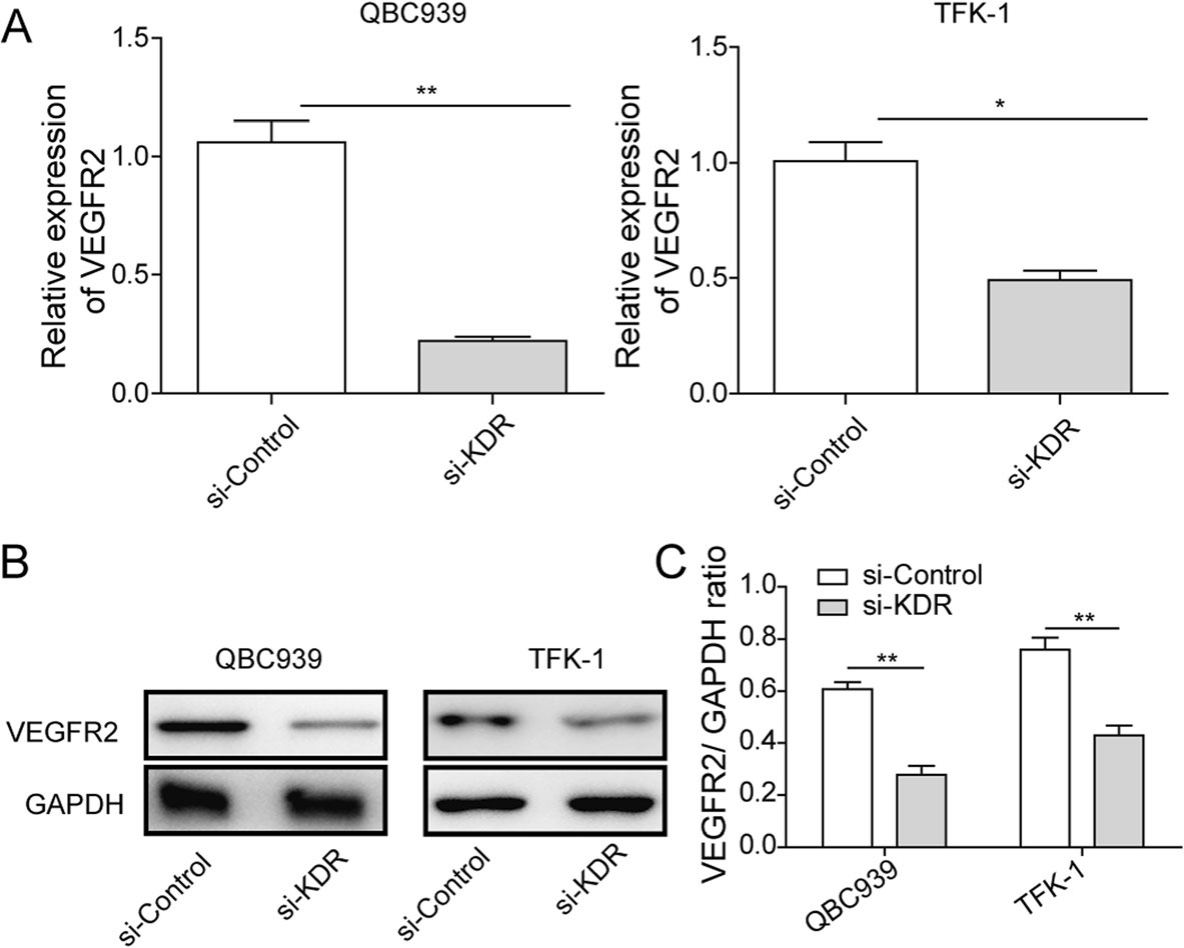
VEGFR2 expression in QBC939 and TFK-1 cells transfected with siKDR or siControl. **a** Cells were transfected with 50 nM siKDR or siControl for 48 h. qRT-PCR was performed to evaluate the mRNA level of VEGFR2 Data shown are means ± SD (*n* = 3). **P < 0.01 and * *P* < 0.05 in QBC939 and TFK-1 cells versus si-Control group, respectively. **b** Protein expression of VEGFR2 in transfected QBC939 and TFK-1 cells were also detected by western-blotting. GAPDH was detected as reference. **c** Densitometric analysis of the autoradiographic plaques of these proteins is shown on the Fig. 1b. Data shown are means ± SD (*n* = 3). **P < 0.01 in QBC939 and TFK-1 cells versus si-Control group

q-PCR and western blotting were performed to investigate the mRNA and protein levels of VEGFR2 in si-KDR or si-Control transfected QBC939 and TFK-1 cells. VEGFR2 mRNA level reduced significantly, showed five and two times lower in siKDR group compared to siControl group in QBC939and TFK-1 cells, respectively (*p* < 0.01; Fig. 1a). Similarly, the protein level also reduced about 2-fold which caused by siKDR (Fig. 1b-c). Both q-PCR and western blotting results suggested KDR interference significantly reduced VEGFR2 expression in both mRNA and protein levels.

### VEGF activated VEGFR2 and promoted proliferation in QBC939 and TFK-1 cells

**Fig. 2:**
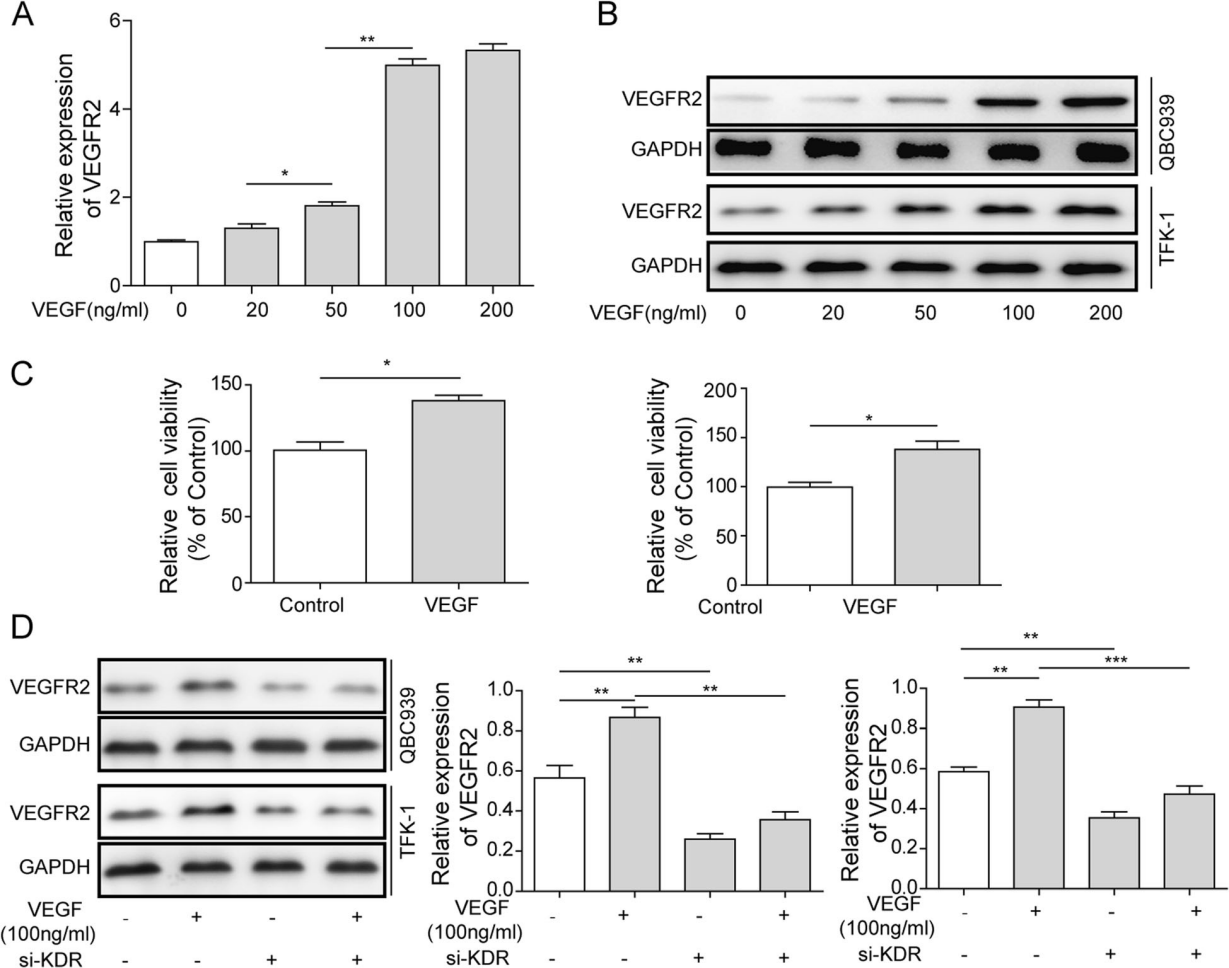
Effects of si-KDR or si-Control on rhVEGF-induced VEGFR2 expression in QBC939 and TFK-1 cells.QBC939 and TFK-1 cells were treated with 0, 20, 50, 100 and 200 ng/ml of rhVEGF for 2 h and obtained from another 24-h incubation in medium, the mRNA level (**a**) and protein expression of VEGFR2 (**b**) was detected. GAPDH was detected as reference. **c** Relative cell viability of QBC939 and TFK-1 cells post 100 ng/ml rhVEGF treatment compared to control group. Data shown are means ± SD (*n* = 3). *P < 0.05 in QBC939 and TFK-1 cells versus control group (not treatment with 100 ng/ml rhVEGF). **d** Protein expression of VEGFR2 in siKDR group with or without 100 ng/ml rhVEGF treatment. GAPDH was detected as reference. Data are representative of three independent experiments. **P < 0.01, ***P < 0.001

After 2 h treatment of 0, 20, 50, 100, or 200 ng/ml rhVEGF, VEGFR2 mRNA and protein level were detected by q-PCR and western blotting. Results showedVEGFR2 mRNA level elevated along the increasing concentration of rhVEGF treatment and reached peak value at 100 ng/ml, which was about 5-fold compared to control group (Fig. 2a). Moreover, VEGFR2 mRNA level kept stable when rhVEGF concentration higher than 100 ng/ml (Fig. 2a). Similarly, the intensity of VEGFR2 protein bands appeared stronger continuously accompany the increasing concentration of rhVEGF treatment and reached strongest at 100 ng/ml rhVEGF (Fig. 2b). These data showed 100 ng/ml rhVEGF is the most suitable concentration for activating VEGFR2.

Following that, MTT assay was performed to show that 100 ng/ml rhVEGF had a significant greater (1.4-fold) enhancement of relative cell viability compared to control (*p* < 0.05; Fig. 2c), suggested that rhVEGF promoted cell viability in QBC939 and TFK-1 cells effectively. In addition, we analyzed the protein level of VEGFR2 by western blot, and found rhVEGF caused a significant increase of VEGFR2 while siKDR caused a significant reduction. However, 100 ng/ml rhVEGF did not significantly reverse the decrease of VEGFR2 in the KDR knockout group (*p* < 0.01; Fig. 2d).

### Apatinib inhibites the migration and invasion of QBC939 and TFK-1 cells

**Fig. 3:**
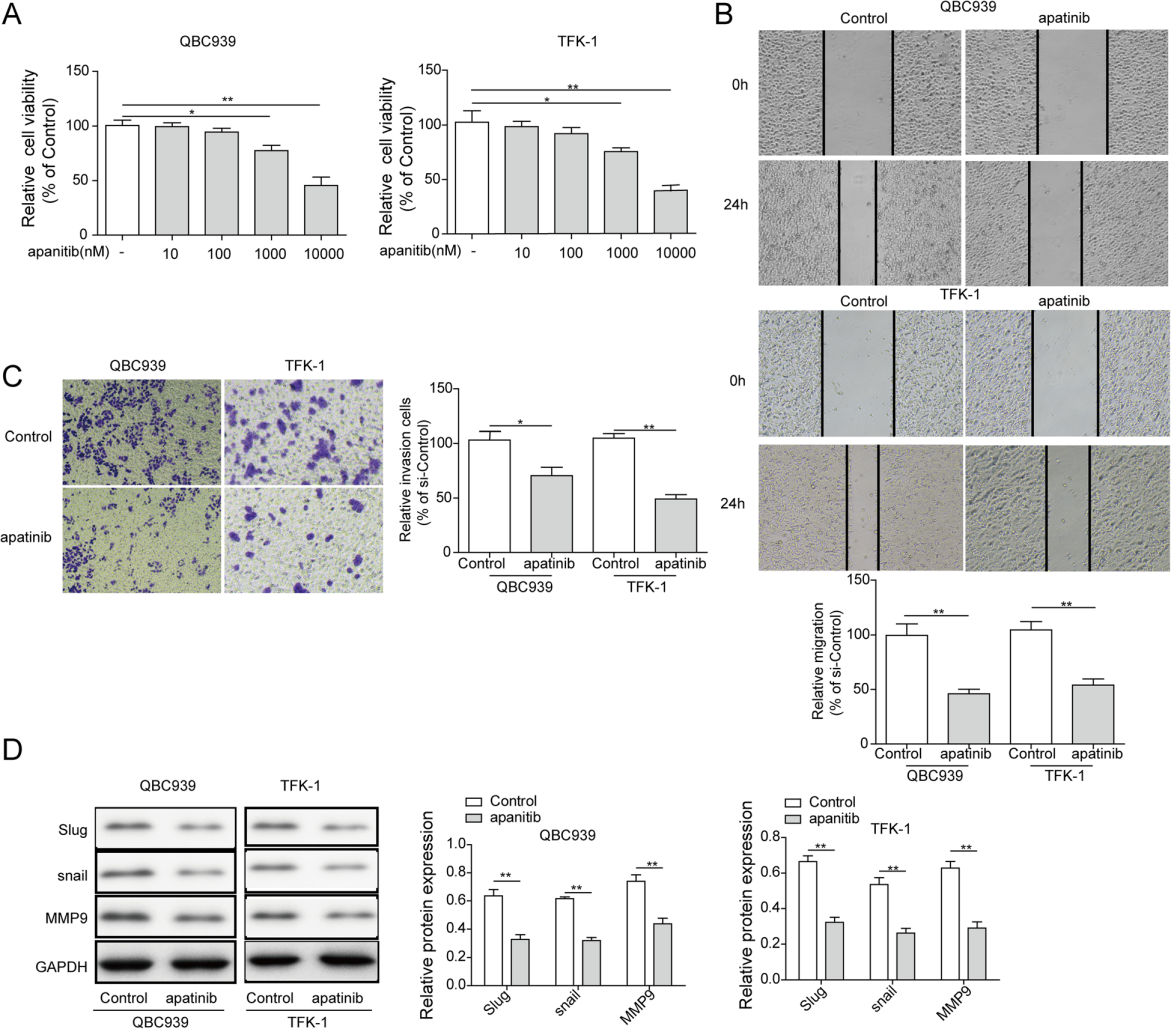
Apatinib inhibit migration and invasion of QBC939 and TFK-1 cells. **a** QBC939 and TFK-1 were treated with apatinib (0, 10, 100, 1000, 10000 nM, respectively) for 48 h. the relative cell viability was detected by MTT assay. Data shown are means ± SD (*n* = 3). *P < 0.05, **P < 0.01 in QBC939 and TFK-1 cells versus control group (0 nM apatinib). **b** Wound healing on QBC939 cells and TFK-1 cells treatment with or without 100 nM apatinibfor 24 h. The migration index (the ratio of migration distance to total distance) was used to measure the movement ability. **c** The cells were treated with apatinib (100 nM) for 24 h. The invasion cells were stained. **d** The cells were treated with apatinib (100 nM) for 24 h. The protein expression of Slug, snail and MMP9 in QBC939 cells and TFK-1 cells were measured by western blot. GAPDH was included as a loading control. *P < 0.05, **P < 0.01 vs control group (0 nM apatinib)

There were no changes of relative cell viability on both QBC939 and TFK-1 cells with 10 and 100 nM apatinib treatment, but 1,000 and 10,000 nM apatinib caused a greatly reduction of relative cell viability compared to control group, suggested 1,000 nM and higher concentration of apatinib could cause cytotoxicity on CCA cells (Fig. 3a). However, we found 100 nM apatinib was enough for CCA cell lines QBC939 and TFK-1 to cause migation and invasion inhibition (*p* < 0.01; Fig. 3b, and *p* < 0.05, *p* < 0.01, respectively; Fig. 3c), Furthermore, metastatic marker Slug, snail and MMP9 protein levels in the cells treated with or without 100 nM apatinib were detected by western blot. Result showed that apatinib could significantly inhibit the protein expression of Slug, snail and MMP9 (Fig. 3d). All these data suggested that apatinib has the effection on inhibiting cell migration and invasion of CCA.

### Apatinib played an essential role on VEGF-mediated migration and invasion in QBC939 and TFK-1 cells

**Fig. 4:**
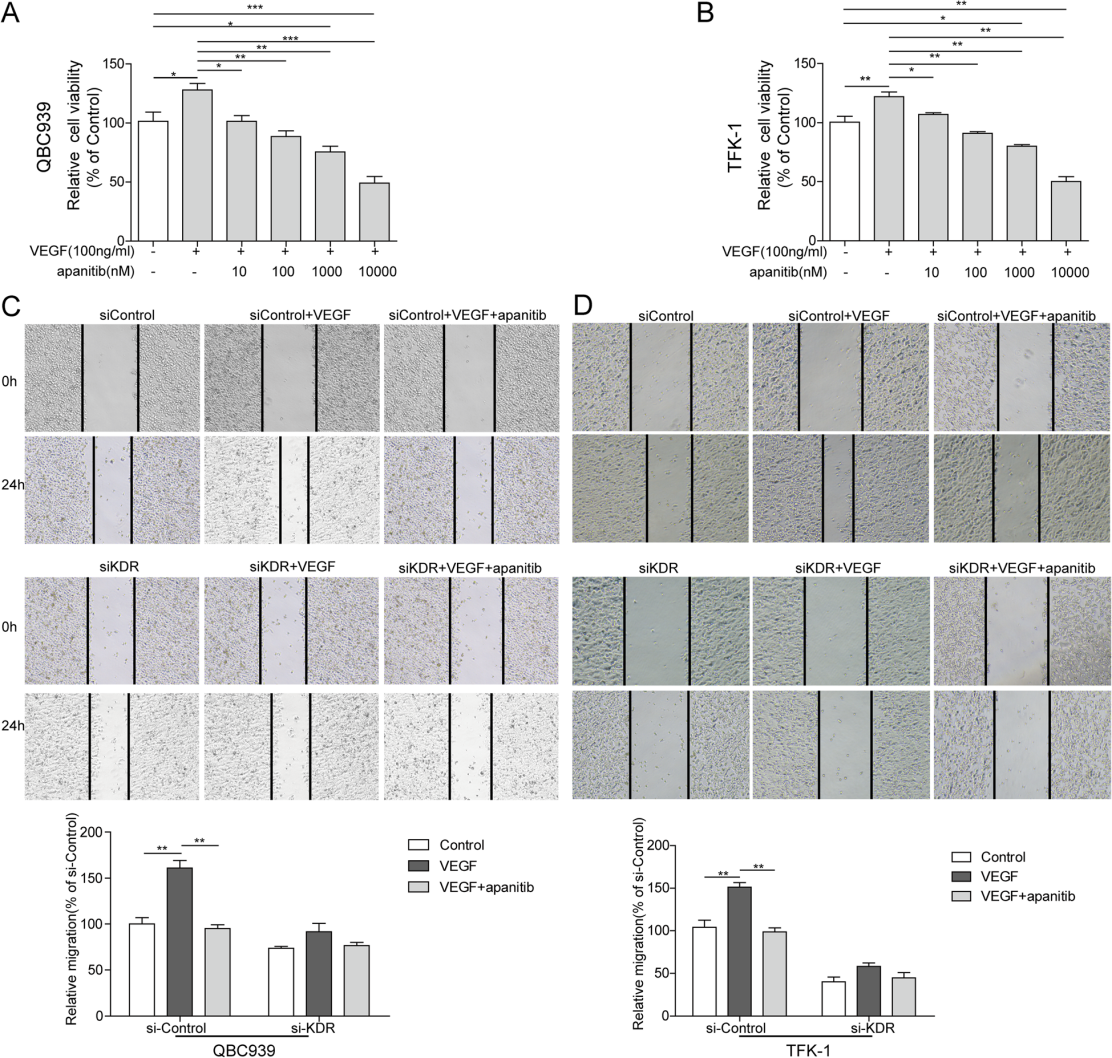
Apatinib inhibits VEGF- induced cell migration and invasion (**a**-**b**) Cell viability of QBC939 (A) and TFK-1 (**b**) cells. Cells were treated with 100 ng/ml rhVEGF for 2 h and then treated with 10, 100, 1,000 and 10,000 nM of apatinib for 24 h. 100 ng/ml rhVEGF significantly increased relative cell viability (compared with 0 ng/ml rhVEGF+ 0 nM apatinib group)and 10–100 nM of apatinib reverses this increase (compared with 100 ng/ml rhVEGF group). Furthermore, 1,000 and 10,000 nM of apatinib inhibite relative cell viability compared with 0 ng/ml rhVEGF+ 0 nM apatinib group. Data are representative of three independent experiments.*P < 0.05,**P < 0.01. **c**-**d** QBC939 (**c**) and TFK-1(**d**) cells migration was measured by wound-healing analysis for 0 and 24 h. si-Control and and si-KDR cells grown in six-well plates were scratched and treated with PBS, VEGF (100 ng/ml), or VEGF (100 ng/ml) combined with apatinib (100 nM) for 24 h. Data are representative of three independent experiments. **P < 0.01

The effect of apatinib on VEGF-mediated cell viability was determined by MTT assay, that total 6 groups were set using increased concentration of apatinib from 0 nM to 10,000 nM with 100 ng/ml rhVEGF. 100 ng/ml rhVEGF significantly increased relative cell viability about 26%compared to control group (*p* < 0.05, *p* < 0.01, respectively Fig. 4a, b). In addition to this, 10 nM and 100 nM apatinib reverses the viability caused by 100 ng/ml VEGF to the normal rate (*p* < 0.05). But 1,000 nM and the higher concentration showed cytotoxicity in both QBC939 and TFK-1 cells (Fig. 4a, b).

**Fig. 5:**
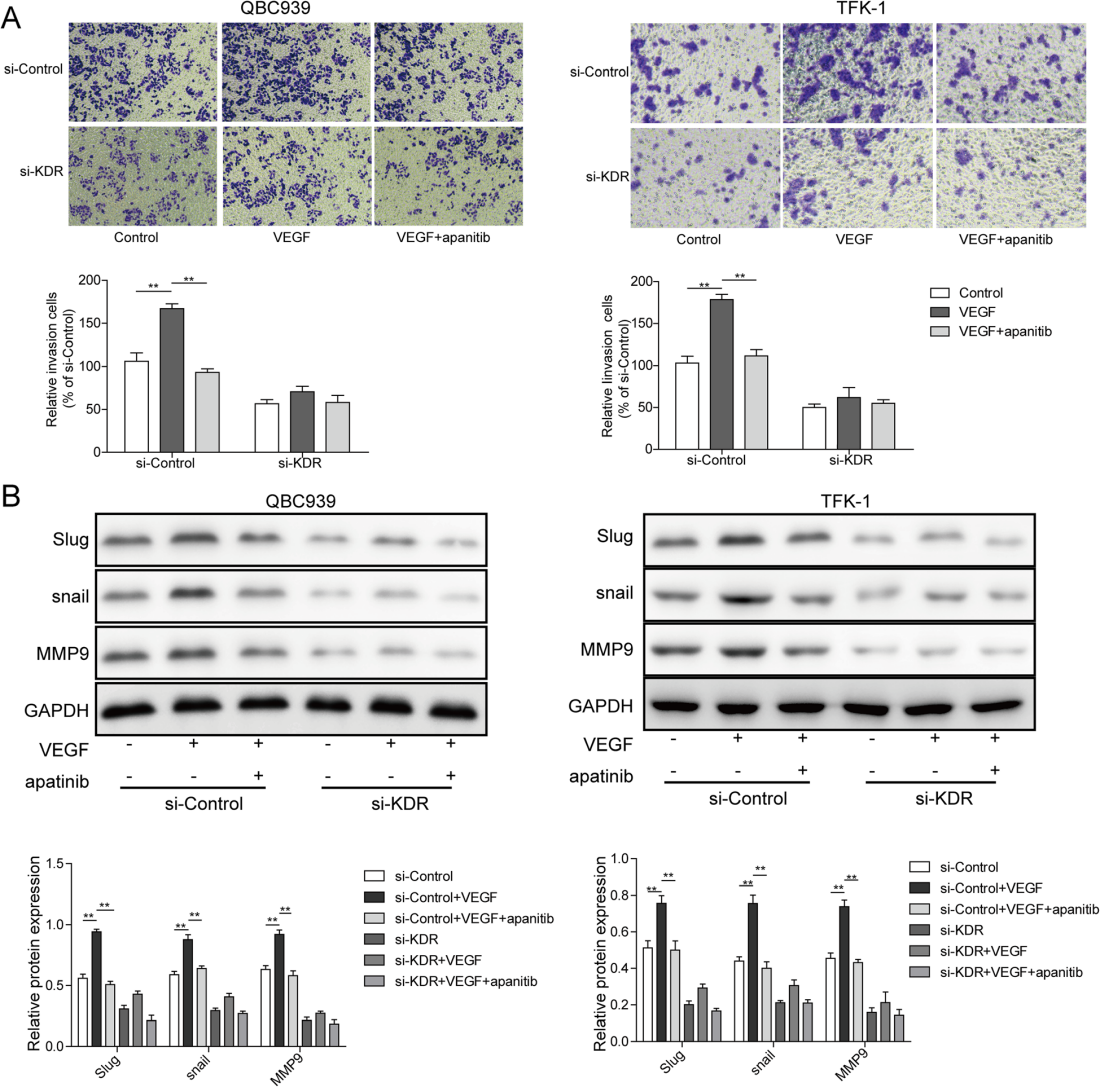
Apatinib inhibits VEGF- induced cell invasion. **a** Representative images of transwell (up) and quantification of invasion cell number (bottom). si-Control and and si-KDR cells grown in six-well plates were scratched and treated with PBS, VEGF (100 ng/ml), or VEGF (100 ng/ml) combined with apatinib (100 nM) for 24 h. Data are representative of three independent experiments. **P < 0.01. **b** Protein expression of Slug, snail and MMP9 in si-Control and si-KDR cells when treated with 100 ng/ml rhVEGF or 100 ng/ml rhVEGF + 100 nM apatinib for 24 h. GAPDH was detected as reference. **P < 0.01

Followed that, wound healing was performed to detect the effect of apatinib (100 nM) on VEGF-mediated QBC939 and TFK-1 cell migration. On siControl group, the wound width significantly reduced 24 h post rhVEGF treatment), while, apatinib treatment suppressed this reduction effectively (*p* < 0.001; Fig. 4c, d). However, on siKDR group, rhVEGF and apatinib treatment showed no significant differenceon wound width as a cause of VEGFR2 knock-down (Fig. 4c, d). These data revealed rhVEGF facilitates QBC939 and TFK-1 cell migration, and apatinib can reverse thiseffect in a VEGFR2 dependent manner.Next, transwell assays were conducted to assess the invasion ability of rhVEGF-induced cells with or without apatinib. On siControl group, rhVEGF significantly promoted the invasion of QBC939 and TFK-1 cells (*p* < 0.01; Fig. 5a), but this invasion was totally suppressed by apatinib (*p* < 0.01; Fig. 5a). However, cells in the rhVEGF and apatinib treating groups had little difference of invasion ability when KDR expression is disturbed (Fig. 5a). Protein levels of metastatic marker slug, snail, MMP9 were also detected, in siControl group, 100 ng/ml rhVEGF significantly promoted the protein expression of slug, snail and MMP9, but 100 nM apatinib dramatically reverse this elevation effect. On the contrary, the protein levels of Slug, snail and MMP9 were stable with rhVEGF and apatinib treatment in the siKDR group (Fig. 5b). These results would reveal that effect of apatinib on AAC cell invasion relying on the presence of VEGFR2.

### Apatinib suppresses VEGF/VEGFR2-mediated signaling through the RAF/MEK/ERK and AKT signaling pathways

**Fig. 6:**
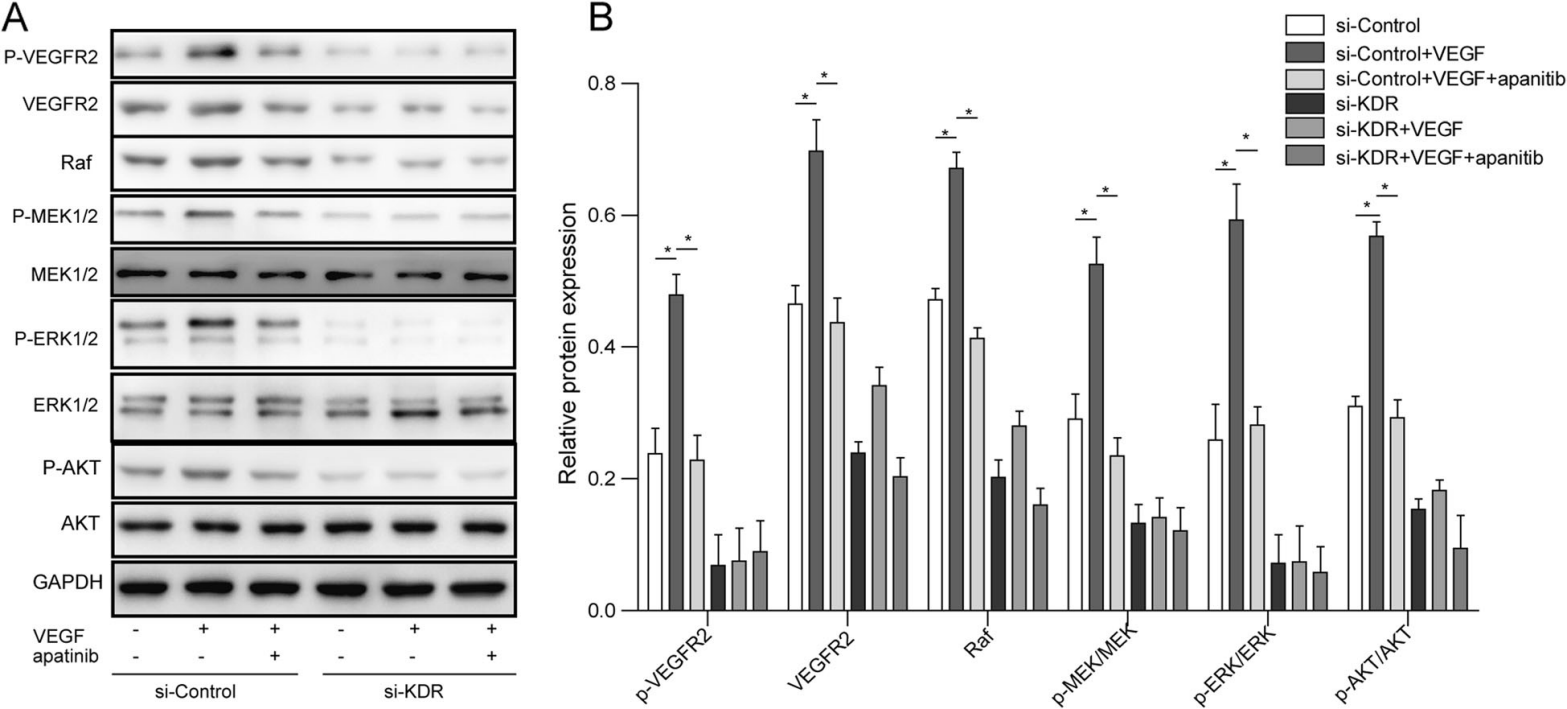
**a** Protein expression of p-VEGFR2, VEGFR2, RAF, p-MEK, MEK, p-ERK1/2, ERK1/2, p-AKT and AKT in transfected QBC939 cells post 100 ng/ml rhVEGF or 100 ng/ml rhVEGF + 100 ng/ml apatinib treatment. GAPDH was detected as reference. **b** Densitometric analysis of the autoradiographic plaques of these proteins is shown on the Fig. 6a . *P < 0.05

Western blotting was performed on siControl and siKDR transfection cells which treated 100 ng/ml rhVEGF with/without 100 nM apatinib, to determine the signaling pathway related to VEGF and its receptor VEGFR2. The expression of p-VEGFR2, VEGR2, RAF, p-MEK, MEK, p-ERK1/2, ERK1/2, p-AKT and AKT were examined, since RAF, MEK and ERK1/2 are the downstream pathway molecules of VEGFR2. Treatment with rhVEGF significantly increased the phosphorylation and total protein of VEGFR2, RAF, phosphorylated MEK, ERK1/2 and AKT protein expression level in siControl group, but had little influence on total protein level of MEK ERK1/2 and AKT (Fig. 6a, b). After blocking VEGFR2 by apatinib, the phosphorylation and total protein level of VEGFR2, phosphorylated MEK and ERK1/2 were reverse to basal level (Fig. 6a, b). On the contrary, both rhVEGF and apatinib treatments had no influence on phosphorylation and total protein level of MEK and ERK1/2 in siKDR group (Fig. 6a, b). Phosphorylation and total protein level of VEGFR2, RAF and phosphorylated AKT were expressed very weak in siKDR group, and this weak expression were stable with or without rhVEGF and apatinib treatment (Fig. 6a, b).

## Discussion

VEGF exerts its biological effects by combining and activating its receptors, which known as VEGFR2 [8, 27]. Publications reveal VEGF plays key role in CCA as the high expression level was detected in the patients’ tumor tissues [1, 15]. Hence, as the antagonist of VEGFR2, apatinib has the potential to become an effective targeted medicine of CCA [16, 19, 20]. In this study, we firstly confirmed the role of VEGF in QBC939 and TFK-1 cells, and then comfirmed the inhibition functions of apatinib in migration and invasion of these two CCA cell lines. Finally, we analyzed the potential signaling pathways Raf/MEk/ERK and PI3K/AKT that might be influenced by apatinib.

Publication reported VEGF could regulate kinds of cancer cells growth through binding to VEGFR [10]. Results in our study showed that exogenous rhVEGF activated the VEGFR2 expression and promoted the cell viability both in QBC939 and TFK-1 cells. It was consistent with publications that blocking VEGF/VEGFR2 pathway have inhibition effect on the growth of cancer cells [7, 14, 32]. Moreover, one paper revealed the role of VEGF in promoting cell growth and inducing the cell apoptosis in CCA [19, 20]. However, whether VEGF was an necessity for tumor migration or invasion in CCA remains unknown.

As the antagonist of VEGFR2, the biological functions of apatinib towards cell migration and invasion in CCA cell lines were performed in this study, and the results provide a first ever comprehensive elucidation of apatinib in anti-CCA progress. We found that it significantly inhibited cell migration and invasion. Moreover, apatinib significantly decreased the expression of metastatic marker such like Slug, snail, and MMP9 in the CCA cell lines. It was consistent with the previous research in lung adenocarcinomas, which found apatinib inhibits cellular invasion and migration by fusion kinase KIF5B-RET via suppressing RET/Src signaling pathway [13]. In addition, it was report intracellular autocrine VEGF signaling promotes EBDC cell proliferation, which can be inhibited by apatinib [19, 20]. And we also found that exogenous rhVEGF significantly promoted migration and invasion in QBC939 and TFK-1 cells, whereas apatinib could reverse these effects. Combine our data and references mentioned above, it exposes the essential role of apatinib in anti-tumour effect, and the apatinib induced inhibition of cell migration and invasion in CCA.

RAF/MEK/ERK pathway has been linked in endothelial cell proliferation [17] and VEGF mediated cell survival [3, 11]. To investigate the possible mechanism of how apatinib works on CCA cells. We found exogenous rhVEGF markedly elevated phosphorylation and total of VEGFR2 protein, and the major downstream targets: phosphorylation of RAF, MEK and ERK1/2, but did not affect the levels of total MEK and ERK1/2. On the contrary, apatinibprominently inhibited this promotion. These results disclosed apatinib could efficiently inhibit the activation of VEGFR2/RAF/MEK/ERK1/2 signal transduction which was induced by VEGF, Besides, we also checked the PI3K/AKT pathway, which was identified as a VEGF related signaling pathway [9, 19, 20, 31]. Same results were gained such like the detection of RAF/MEK/ERK1/2 pathway, which provided another molecular mechanism of apatinib acted on CCA cells. These results are supported by several studies that the anti-apoptosis effect of VEGF closely related to PI3K/AKT/mTOR signaling pathway [9, 19, 20, 31]. Previous studies has revealed that both VEGF/MEK/ERK and PI3K/AKT pathways play key role in developing CCA [9, 24, 25, 30]. Here, we found that apatinib could reverse the rhVEGF induced cell migration and invasion by blocking these two pathways. Combined with our data and references we have mentioned above, apatinib has an important role in CCA migration and invasion, and apatinib exerts excellent anti-tumor function in CCA cell lines. However, whether apatinib could perform the same antitumor function of CCA in vivo still needs to be studied. Additionally, since angiogenesis of endothelial cells is an important factor in promoting tumor metastasis, and apatinib might play key role in angiogenesis via VEGFR2 as it expressed in endothelial cells. This would be worth to study in our future work.

## Conclusions

In conclusion, our study demonstrates that apatinib inhibits VEGF-mediated cell migration and invasion of CCA cell lines, possibly by blocking VEGFR2-dependent RAF/MEK/ERK and PI3K/AKT pathways. Our article shows the migration and invasion inhibition effect of apatinib acting directly on the CCA cell lines for the first time, hoping to attract more researchers’ attention on deeper understanding and evaluating the potential clinical utility of apatinib.

## Abbreviations

CCA: Cholangiocarcinoma
KDR: Kinase insert domain receptor, also known as VEGFR2
rhVEGF: Recombined human VEGF
siKDR: Small interference KDR
VEGF: Vascular endothelial growth factor
VEGFR2: VEGF receptor 2

## References

[1] Abdel-Razik A, ElMahdy Y, Hanafy EE, Elhelaly R, Elzehery R, Tawfik AM, Eldars W (2016). Insulin-like growth Factor-1 and vascular endothelial growth factor in malignant and benign biliary obstructions. Am J Med Sci.

[2] Amo Y, Masuzawa M, Hamada Y, Katsuoka K (2004). Serum concentrations of vascular endothelial growth factor-D in angiosarcoma patients. Br J Dermatol.

[3] Berra E, Milanini J, Richard DE, Le GM, Viñals F, Gothié E, Roux D, Pagès G, Pouysségur J (2000). Signaling angiogenesis via p42/p44 MAP kinase and hypoxia. Biochem Pharmacol.

[4] Callea F, Sergi C, Fabbretti G, Brisigotti M, Cozzutto C, Medicina D (2010). Precancerous lesions of the biliary tree. J Surg Oncol.

[5] Chatterjee S, Heukamp LC, Siobal M, Schottle J, Wieczorek C, Peifer M, Frasca D, Koker M, Konig K, Meder L, Rauh D, Buettner R, Wolf J, Brekken RA, Neumaier B, Christofori G, Thomas RK, Ullrich RT (2013). Tumor VEGF:VEGFR2 autocrine feed-forward loop triggers angiogenesis in lung cancer. J Clin Invest.

[6] Deoliveira ML, Cunningham SC, Cameron JL, Kamangar F, Winter JM, Lillemoe KD, Choti MA, Yeo CJ, Schulick RD (2007). Cholangiocarcinoma: thirty-one-year experience with 564 patients at a single institution. Ann Surg.

[7] Gaudio E, Barbaro B, Alvaro D, Glaser S, Francis H, Ueno Y, Meininger CJ, Franchitto A, Onori P, Marzioni M, Taffetani S, Fava G, Stoica G, Venter J, Reichenbach R, De Morrow S, Summers R, Alpini G (2006). Vascular endothelial growth factor stimulates rat cholangiocyte proliferation via an autocrine mechanism. Gastroenterology.

[8] Eremina V, Quaggin SE (2004). The role of VEGF-A in glomerular development and function. Curr Opin Nephrol Hypertens.

[9] Ewald F, Norz D, Grottke A, Hofmann BT, Nashan B, Jucker M (2014). Dual inhibition of PI3K-AKT-mTOR- and RAF-MEK-ERK-signaling is synergistic in cholangiocarcinoma and reverses acquired resistance to MEK-inhibitors. Investig New Drugs.

[10] Ferrara N, Gerber HP, Lecouter J (2003). The biology of VEGF and its receptors. Nat Med.

[11] Gupta K, Kshirsagar S, Li W, Gui L, Ramakrishnan S, Gupta P, Law PY, Hebbel RP (1999). VEGF prevents apoptosis of human microvascular endothelial cells via opposing effects on MAPK/ERK and SAPK/JNK signaling. Exp Cell Res.

[12] Li YY, Li H, Lv P, Liu G, Li XR, Tian BN, Chen DJ (2011). Prognostic value of cirrhosis for intrahepatic cholangiocarcinoma after surgical treatment. J Gastrointest Surg.

[13] Lin C, Wang S, Xie W, Zheng R, Gan Y, Chang J (2016). Apatinib inhibits cellular invasion and migration by fusion kinase KIF5B-RET via suppressing RET/Src signaling pathway. Oncotarget.

[14] Liu Y, Qiao Y, Hu C, Liu L, Zhou L, Liu B, Chen H, Jiang X (2016). VEGFR2 inhibition by RNA interference affects cell proliferation, migration, invasion, and response to radiation in Calu-1 cells. Clinical & translational oncology.

[15] Lv L, Wei M, Lin P, Chen Z, Gong P, Quan Z, Tang Z (2017). Integrated mRNA and lncRNA expression profiling for exploring metastatic biomarkers of human intrahepatic cholangiocarcinoma. Am J Cancer Res.

[16] Ma FC, Yu Q, Zeng ZM, He RQ, Mo CH, Zhong JC, Ma J, Feng ZB, Chen G, Hu XH (2017). Progression-free survival of up to 8 months of an advanced intrahepatic cholangiocarcinoma patient treated with apatinib: a case report. Onco Targets Ther.

[17] Meadows KN, Bryant P, Pumiglia K (2001). Vascular endothelial growth factor induction of the angiogenic phenotype requires Ras activation. J Biol Chem.

[18] Park BK, Paik YH, Park JY, Park KH, Bang S, Park SW, Chung JB, Park YN, Song SY (2006). The clinicopathologic significance of the expression of vascular endothelial growth factor-C in intrahepatic cholangiocarcinoma. Am J Clin Oncol.

[19] Peng H, Zhang Q, Li J, Zhang N, Hua Y, Xu L, Deng Y, Lai J, Peng Z, Peng B, Chen M, Peng S, Kuang M (2016). Apatinib inhibits VEGF signaling and promotes apoptosis in intrahepatic cholangiocarcinoma. Oncotarget.

[20] Peng S, Zhang Y, Peng H, Ke Z, Xu L, Su T, Tsung A, Tohme S, Huang H, Zhang Q, Lencioni R, Zeng Z, Peng B, Chen M, Kuang M (2016). Intracellular autocrine VEGF signaling promotes EBDC cell proliferation, which can be inhibited by Apatinib. Cancer Lett.

[21] Ramirez-Merino N, Aix SP, Cortes-Funes H (2013). Chemotherapy for cholangiocarcinoma: an update. World J Gastrointest Oncol.

[22] Rizvi S, Gores GJ (2013). Pathogenesis, diagnosis, and management of cholangiocarcinoma. Gastroenterology.

[23] Senger DR, Galli SJ, Dvorak AM, Perruzzi CA, Harvey VS, Dvorak HF (1983). Tumor cells secrete a vascular permeability factor that promotes accumulation of ascites fluid. Science.

[24] Shroff RT, Yarchoan M, O'Connor A, Gallagher D, Zahurak ML, Rosner G, Ohaji C, Sartorius-Mergenthaler S, Subbiah V, Zinner R, Azad NS (2017). The oral VEGF receptor tyrosine kinase inhibitor pazopanib in combination with the MEK inhibitor trametinib in advanced cholangiocarcinoma. Br J Cancer.

[25] Simone Valeria, Brunetti Oronzo, Lupo Luigi, Testini Mario, Maiorano Eugenio, Simone Michele, Longo Vito, Rolfo Christian, Peeters Marc, Scarpa Aldo, Azzariti Amalia, Russo Antonio, Ribatti Domenico, Silvestris Nicola (2017). Targeting Angiogenesis in Biliary Tract Cancers: An Open Option. International Journal of Molecular Sciences.

[26] Tang D, Nagano H, Yamamoto H, Wada H, Nakamura M, Kondo M, Ota H, Yoshioka S, Kato H, Damdinsuren B (2006). Angiogenesis in cholangiocellular carcinoma: expression of vascular endothelial growth factor, angiopoietin-1/2, thrombospondin-1 and clinicopathological significance. Oncol Rep.

[27] Terman BI, Dougher-Vermazen M, Carrion ME, Dimitrov D, Armellino DC, Gospodarowicz D, Bohlen P (1992). Identification of the KDR tyrosine kinase as a receptor for vascular endothelial cell growth factor. Biochem Biophys Res Commun.

[28] Tian S, Quan H, Xie C, Guo H, Lu F, Xu Y, Li J, Lou L (2011). YN968D1 is a novel and selective inhibitor of vascular endothelial growth factor receptor-2 tyrosine kinase with potent activity in vitro and in vivo. Cancer Sci.

[29] Tsuzuki T, Ogata Y, Iida S, Nakanishi I, Takenaka Y, Yoshii H (1983). Carcinoma of the bifurcation of the hepatic ducts. Arch Surg.

[30] Wiedmann MW, Mossner J (2010). Molecular targeted therapy of biliary tract cancer--results of the first clinical studies. Curr Drug Targets.

[31] Xu D, Ma Y, Zhao B, Li S, Zhang Y, Pan S, Wu Y, Wang J, Wang D, Pan H, Liu L, Jiang H (2014). Thymoquinone induces G2/M arrest, inactivates PI3K/Akt and nuclear factor-kappaB pathways in human cholangiocarcinomas both in vitro and in vivo. Oncol Rep.

[32] Zhang L, Wang JN, Tang JM, Kong X, Yang JY, Zheng F, Guo LY, Huang YZ, Zhang L, Tian L (2012). VEGF is essential for the growth and migration of human hepatocellular carcinoma cells. Mol Biol Rep.

